# *P-HYDROXYPHENYLPYRUVATE DIOXYGENASE* from *Medicago sativa* is involved in vitamin E biosynthesis and abscisic acid-mediated seed germination

**DOI:** 10.1038/srep40625

**Published:** 2017-01-13

**Authors:** Jishan Jiang, Zhihong Chen, Liping Ban, Yudi Wu, Jianping Huang, Jinfang Chu, Shuang Fang, Zan Wang, Hongwen Gao, Xuemin Wang

**Affiliations:** 1 Institute of Animal Sciences, Chinese Academy of Agricultural Sciences, Beijing 100193, China; 2 National Animal Husbandry Service, Ministry of Agriculture, Beijing 100125, China; 3 College of Animal Science and Technology, China Agricultural University, Beijing 100193, China; 4 National Centre for Plant Gene Research, Institute of Genetics and Developmental Biology, Chinese Academy of Sciences, Beijing 100101, China

## Abstract

*P-HYDROXYPHENYLPYRUVATE DIOXYGENASE (HPPD)* is the first committed enzyme involved in the biosynthesis of vitamin E, and is characterized by catalyzing the conversion of p-hydroxyphenyl pyruvate (HPP) to homogentisic acid (HGA). Here, an *HPPD* gene was cloned from *Medicago sativa* L. and designated *MsHPPD*, which was expressed at high levels in alfalfa leaves. PEG 6000 (polyethylene glycol), NaCl, abscisic acid and salicylic acid were shown to significantly induce *MsHPPD* expression, especially in the cotyledons and root tissues. Overexpression of *MsHPPD* was found to significantly increase the level of β-tocotrienol and the total vitamin E content in *Arabidopsis* seeds. Furthermore, these transgenic *Arabidopsis* seeds exhibited an accelerated germination time, compared with wild-type seeds under normal conditions, as well as under NaCl and ABA treatments. Meanwhile, the expression level of several genes associated with ABA biosynthesis (*NCED3, NCED5* and *NCED9*) and the ABA signaling pathway (*RAB18, ABI3* and *ABI5*) were significantly down-regulated in *MsHPPD-*overexpressing transgenic lines, as well as the total free ABA content. Taken together, these results demonstrate that *MsHPPD* functions not only in the vitamin E biosynthetic pathway, but also plays a critical role in seed germination via affecting ABA biosynthesis and signaling.

Vitamin E is an essential nutrient for animals and humans, the physiological significance of this substance has been studied widely. Specifically, vitamin E has been shown to scavenge singlet oxygen^1^, reduce lipid oxidation by-products and inhibit lipid peroxidation^2^, thereby helping plants to defend against various stresses and extending the shelf life of meat via keeping it fresh^3^. Furthermore, vitamin E deficiency has been shown to result in infertility and fetal death in animals^4^,^5^. In contrast, sufficient uptake of vitamin E in human and animal diets leads to numerous benefits, such as a decreased risk of select cancers and atherosclerosis, a bolstering of the immune system, and a reduction in instances of various vision maladies^6^.

Vitamin E is not a single compound, but rather the collective name for a group of eight lipid-soluble antioxidants consisting of a polar chromanol head group and a hydrophobic prenyl tail^7^, which are derived from the methylerythritol-4-phosphate (MEP) and shikimate pathways. Four of these compounds are termed tocopherols, and the other four are termed tocotrienols, and depending on the saturation level of the hydrophobic tail and also the number and position of the methyl groups on the chromanol ring, members of the vitamin E group are classified into α-, β-, γ- and δ- forms^8^. The plastidial aromatic amino acid metabolism pathway is utilized for the synthesis of the tocopherol and tocotrienol head group (homogentisic acid, HGA) and the deoxyxylulose-5-phosphate pathway is used for the synthesis of the hydrophobic tail (either phytyl-PP for the tocopherols or GGDP for the tocotrienols). Vitamin E is only synthesized in higher plants and other oxygen-evolving phototrophs, including some cyanobacteria and all species of green algae^9^. In plants, the production of HGA is the first step in tocopherol synthesis, and HGA is synthesized from p-hydroxyphenyl pyruvate (HPP) in a reaction catalyzed by HPPD. Ergo, HPPD is essential for plant viability, and mutant plants with null alleles of *HPPD* exhibit a lethal photobleaching phenotype^10^. It has also been shown previously that overexpression of barley *HPPD* results in a two-fold increase to the vitamin E content of transgenic tobacco seeds^11^. Furthermore, overexpression of *AtHPPD* increased the total vitamin E level by seven-fold in *Synechocystis*^12^, and significantly increased vitamin E accumulation in transgenic potato tubers^13^. Collectively, these observations indicate that modifying *HPPD* gene expression is a valid strategy to utilize during attempts to modulate the total vitamin E content of plant tissues.

The growth inhibitor abscisic acid (ABA) is widely recognized as an important phytohormone involved in plant stress response and seed germination^14^. It accumulates most notably in dry seeds and declines rapidly subsequent to seed germination^15^. ABA is formed via the cleavage of C40 carotenoids, which originate from the MEP pathway^16^,^17^ and share a common condensed GGDP intermediate with vitamin E. Isopentenyl pyrophosphate (IPP), which is synthesized from the MEP and/or Mevalonate (MVA) pathways, is subjected to a cascade of reactions, before ultimately being condensed to form geranylgeranyl diphosphate (GGDP), which has been shown to be a key intermediate in the synthesis of carotenoids and tocochromanols^18^. With regards to ABA, 9-*cis*-neoxanthin, which is formed during the course of the carotenoid biosynthetic pathway, is cleaved by 9-cis-epoxycarotenoid dioxygenase (*NCED*) genes to form xanthoxin, which is ultimately modified to ABA^16^. Nine *NCED* genes have been identified in *Arabidopsis*, amongst which *NCED 2, 3, 5, 6,* and *9* are thought to play principal roles in determining the ABA content^19^. There are also a number of genes involved in the ABA signaling pathway, and *ABSISIC ACID INSENSITIVE 3 (ABI3)* and *ABSISIC ACID INSENSITIVE 5 (ABI5)* have been shown to play important roles pertaining to seed germination^20^,^21^.

Alfalfa, as an important perennial leguminous forage crop, has multiple agro-ecological advantages over other crop plants, including protecting soil from erosion, as well as fixing and providing nitrogen for neighboring plants^22^,^23^. Additionally, alfalfa is considered an important feedstock that provides vitamins, proteins, and minerals to animals. However, alfalfa is a perennial autotetraploid, and its cross-pollinated genetic background has classically restricted the discovery and application of novel gene resources in alfalfa^24^. Vitamin E biosynthesis has been widely studied in other plants; however, little is known about the genes involved in vitamin E biosynthesis in forage crops. As such, discovering and characterizing related genes will enrich our knowledge on the biosynthetic mechanisms of this essential nutrient in forage crops, including alfalfa.

In this study, we identified an *HPPD* gene in alfalfa, determined that that it is phylogentically closest to *MtHPPD* from *Medicago truncatula.* The expression of *MsHPPD* was induced by different stress conditions in alfalfa, and overexpression of *MsHPPD* increased the vitamin E content in *Arabidopsis* seeds. The germination of these transgenic *Arabidopsis* seeds was accelerated, as compared to wild type seeds, under normal growth conditions. Moreover, seeds from transgenic *Arabidopsis* were more resistant to salt stress and less sensitive to ABA treatment, manifesting in the transgenic seeds having an elevated germination rate. Ultimately, in addition to its role in vitamin E accumulation, we showed that *MsHPPD* plays a positive role in seed germination via regulating ABA biosynthesis and the subsequent ABA signaling pathway.

## Results

### Cloning and sequence analysis of the *MsHPPD* gene from *Medicago sativa*

**Figure 1:**
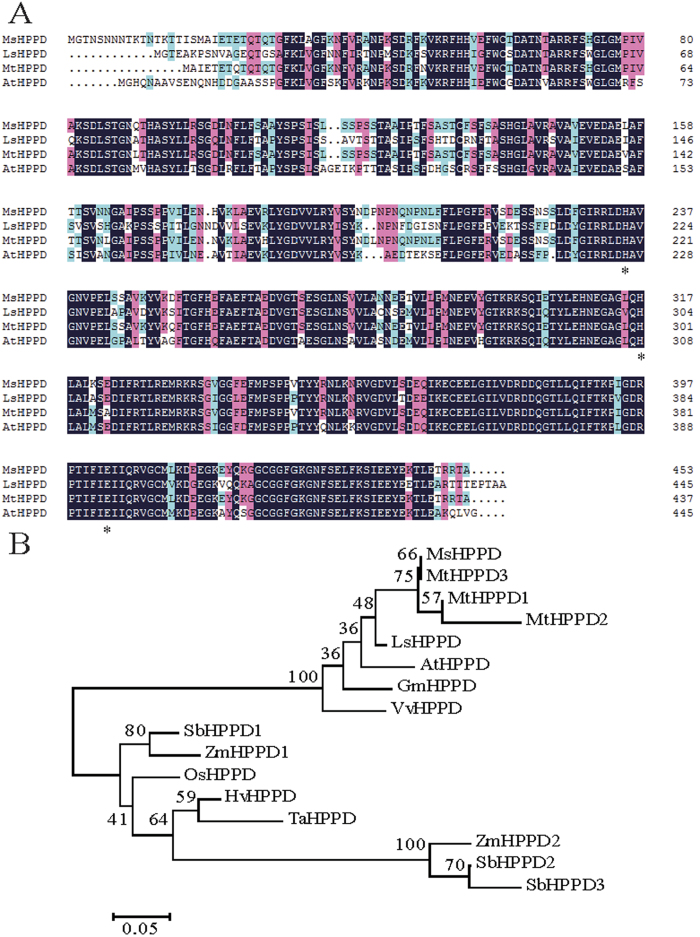
Bioinformatic analysis of the *MsHPPD* sequence. (**A**) Multiple sequence alignment of plant HPPDs. MsHPPD: *Medicago sativa*; LsHPPD: *Lactuca sativa* (ACN78586.1); MtHPPD: *Medicago truncatula* (XP_003617391.1); AtHPPD: *Arabidopsis thaliana* (CBI85437.1). Asterisk: Fe binding sites. Colors highlighted homology levels. Black represents identity =100%, red represents identity ≥75%, green represents identity ≥50%. (**B**) Phylogenetic tree of MsHPPD and HPPDs from other plant species. MEGA 6.0 was used to construct the tree using the neighbor-joining method, bootstrap =1000. Protein sequences of HPPD were downloaded from NCBI as follows: MtHPPD1: *Medicago truncatula* (XP_003617384.1); MtHPPD2: *Medicago truncatula* (XP_003617382.2); MtHPPD3: *Medicago truncatula* (XP_003617391.1); LsHPPD: *Lactuca sativa* (ACN78586.1); AtHPPD: *Arabidopsis thaliana* (CBI85437.1); VvHPPD: *Vitis vinifera* (CAN71143.1); GmHPPD: *Glycine max* (ABQ96868.1); OsHPPD: *Oryza sativa* (EAZ21880.1); ZmHPPD1: *Zea mays*: (NP_001105782.1); ZmHPPD2: *Zea mays*: (XP_008653702.1); SbHPPD1: *Sorghum bicolor* (XP_002453359.1); SbHPPD2: *Sorghum bicolor* (XP_002461829.1); SbHPPD3: *Sorghum bicolor* (XP_002461838.1); TaHPPD: *Triticum aestivum* (CAJ29893.1); HvHPPD: *Hordeum vulgare* (CBI85441.1).

Using the known sequence of *Medicago truncatula HPPD* gene, a conserved 648-bp fragment was cloned from alfalfa. Rapid amplification of cDNA ends (RACE) was performed based on this conserved sequence, and a 2064-bp full-length *HPPD* gene was obtained by combining the1188-bp and 1001-bp fragments isolated by 3′ RACE and 5′ RACE, respectively. This full-length sequence contains a 1305-bp open reading frame, which encodes a protein of 434 amino acids. Multiple protein sequence alignment revealed that the protein sequence was most closely related to known HPPD protein sequences from other organisms, which belong to the Glo_EDI_BRP_ like superfamily, and contain an HPPD-N-like and an HPPD-C-like domain. Additionally, three iron (Fe^2+^) binding sites, which are essential for the activity of *HPPD*, are also conserved among all the HPPD sequences (Fig. 1A), including in the alfalfa sequence. Thus, the identified gene from alfalfa was designated *MsHPPD* (accession number KY081399). To investigate the evolutionary relationships between *MsHPPD* and HPPD homologs from other species, phylogenetic analyses were performed with MEGA6.0 using the Neighbor-joining method^25^. This showed that HPPDs from monocotyledonous and dicotyledonous species grouped into two separate clades. As expected, MsHPPD clusters together with the eudicotyledonous sequences, including *MtHPPD* from *Medicago truncatula, LsHPPD* from *Lactuca sativa,* and *AtHPPD* from *Arabidopsis thaliana* (Fig. 1B).

### *MsHPPD* expression in different tissues

**Figure 2:**
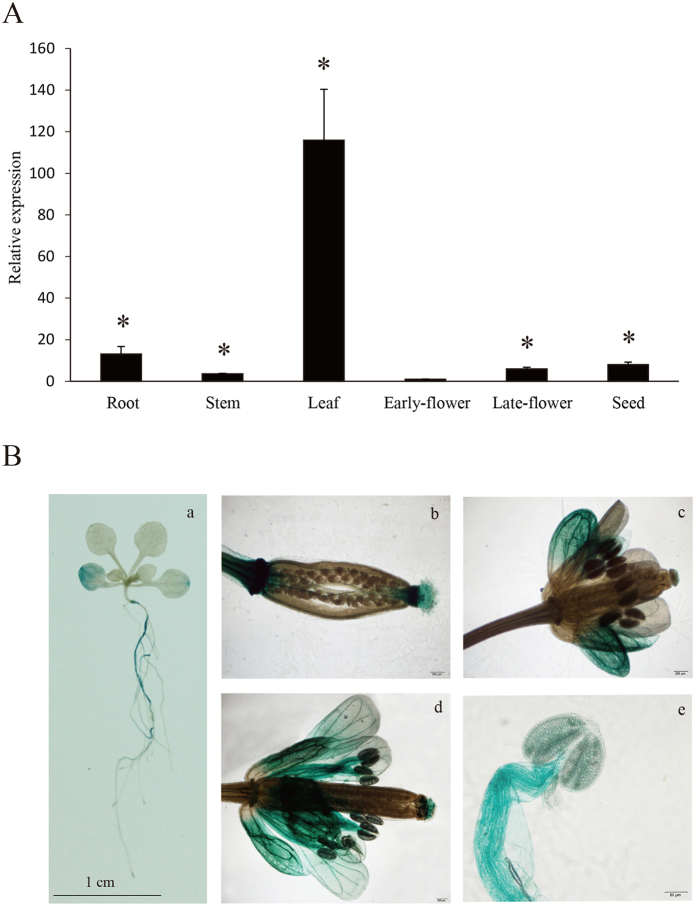
Expression pattern analysis of *MsHPPD* gene. (**A**) Expression levels of *MsHPPD* in different organs of alfalfa. RNA was extracted from tissues collected from two-year-old alfalfa. Data presented are mean ± SD, each with three biological replicates and three technical replicates. Expression levels are relative to early flower; Statistical analyses were carried out via a two-tailed Student’s *t-*test, asterisks show the significance of P < 0.05. (**B**) Histochemical GUS staining of pHPPD::GUS lines. (a) Ten-day-old *Arabidopsis* seedling, bar: 1 cm; (b) Silique of transgenic *Arabidopsis*, bar: 200 μm; (c) Early flower, bar: 200 μm; (d) Late flower, bar: 200 μm; (e) Pollen, bar: 50 μm.

Quantitative reverse transcription-PCR (qRT-PCR) was performed to determine the expression pattern of *MsHPPD* in different organs of *M. sativa*. The results showed *MsHPPD* transcripts were detectable in all tested organs, with the highest expression in rosette leaves and the lowest expression in early flowers (Fig. 2A). This implies that *MsHPPD* may play a more active role in leaves.

To further confirm the transcriptional expression pattern of *MsHPPD*, a pHPPD::GUS construct was transformed into *Arabidopsis*. GUS expression was detected in cotyledons, primary roots, sepals, petals, stigma, filament tubes, pollens, and the ends of seeds in transgenic lines. However, no GUS activity was observed in the root tip or hypocotyls (Fig. 2B).

### *MsHPPD* expression under various conditions

**Figure 3:**
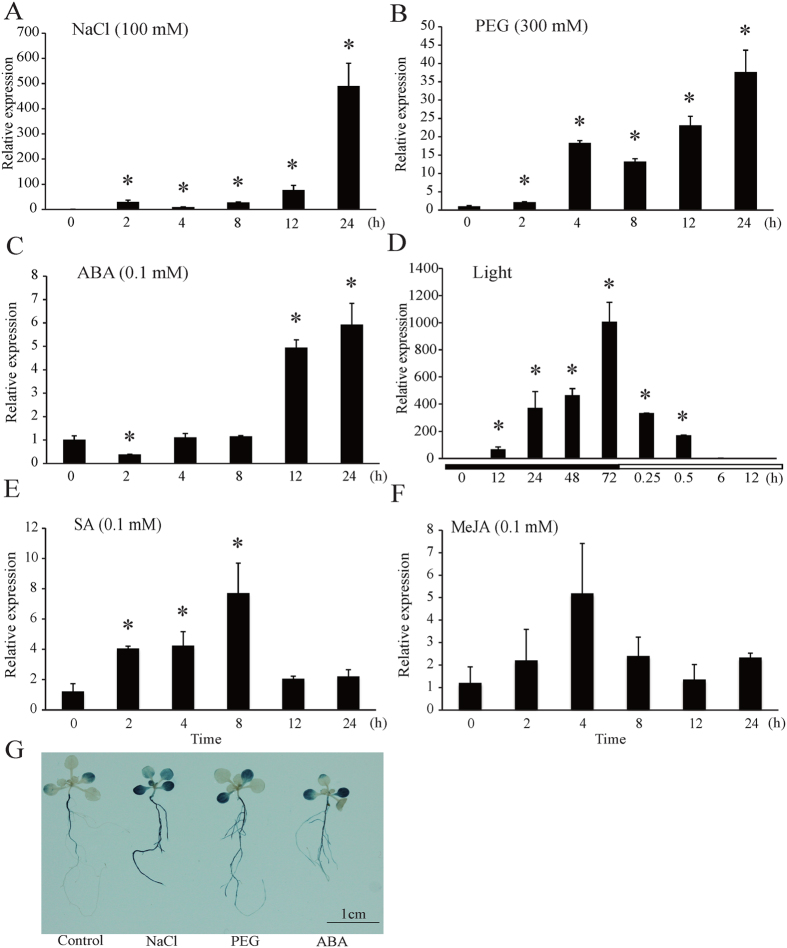
Expression profiles of *MsHPPD* under various treatments. (**A**) Expression levels of *MsHPPD* under NaCl treatment. (**B**) Expression levels of *MsHPPD* under PEG treatment. (**C**) Expression levels of *MsHPPD* under ABA treatment. (**D**) Expression levels of *MsHPPD* under dark to light transduction treatment. (**E**) Expression levels of *MsHPPD* under SA treatment. (**F**) Expression levels of *MsHPPD* under MeJA treatment. Two-week-old alfalfa seedlings were treated with NaCl (100 mM), PEG (300 mM), ABA (0.1 mM), dark/light, SA (0.1 mM) and MeJA (0.1 mM). Samples were collected at each time point, with three biological replicates. Data are presented as mean ± SD, each with three biological replicates and three technical replicates. Statistical analyses were carried out via a two-tailed Student’s *t-*test with a significance of P < 0.05. (**G**) Histochemical GUS staining of pHPPD::GUS under various conditions. Two-week-old *Arabidopsis* seedlings were treated with NaCl (100 mM), PEG (300 mM) and ABA (0.1 mM) for 24 h and then stained with GUS. Bar: 1 cm.

The promoter sequence of *MsHPPD* was analyzed using PlantCARE (http://bioinformatics.psb.ugent.be/webtools/plantcare/html/) to better investigate the expressional regulation of *MsHPPD*^26^. The results revealed *cis*-elements, which have been shown to respond to defense and stress signals including salicylic acid, gibberellin, and light signals are presented in the promoter region of *MsHPPD* (Fig. S1). To explore the possible roles of these *cis*-elements with regard to regulating the expression of *MsHPPD*, expressional analysis of *MsHPPD* was executed under various stress treatments. These results showed that *MsHPPD* transcripts were gradually up-regulated in seedlings treated with increasing treatment period when seedlings were exposed to NaCl, PEG, and ABA (Fig. 3A–C). Most notably, the expression of *MsHPPD* increased 500-fold after a 24 h treatment with NaCl, relative to the levels seen in the mock treatment. Likewise, treatments utilizing extended periods of darkness revealed that the expression of *MsHPPD* rose gradually as the darkness treatment time increased; however, upon exposure to light, the expression levels of *MsHPPD* dropped sharply, and this decaying trend continued with prolonged exposure to light (Fig. 3D). The expression levels of *MsHPPD* increased moderately, albeit significantly, subsequent to salicylic acid treatment, until the 8 h time point, at which point *MsHPPD* expression returned to the control level (Fig. 3E). No significant differences were observed after methyl jasmonic acid treatment (Fig. 3F).

pHPPD::GUS was used to approximate the expression levels of *MsHPPD* in response to NaCl, PEG, and ABA treatments. Seedlings treated with NaCl, PEG, or ABA showed increase GUS activity across the plants, especially in the roots and cotyledons. This observation is consistent with the qRT-PCR results (Fig. 3G).

### Expression of *MsHPPD* and endogenous vitamin E pathway genes in transgenic *Arabidopsis*

**Figure 4:**
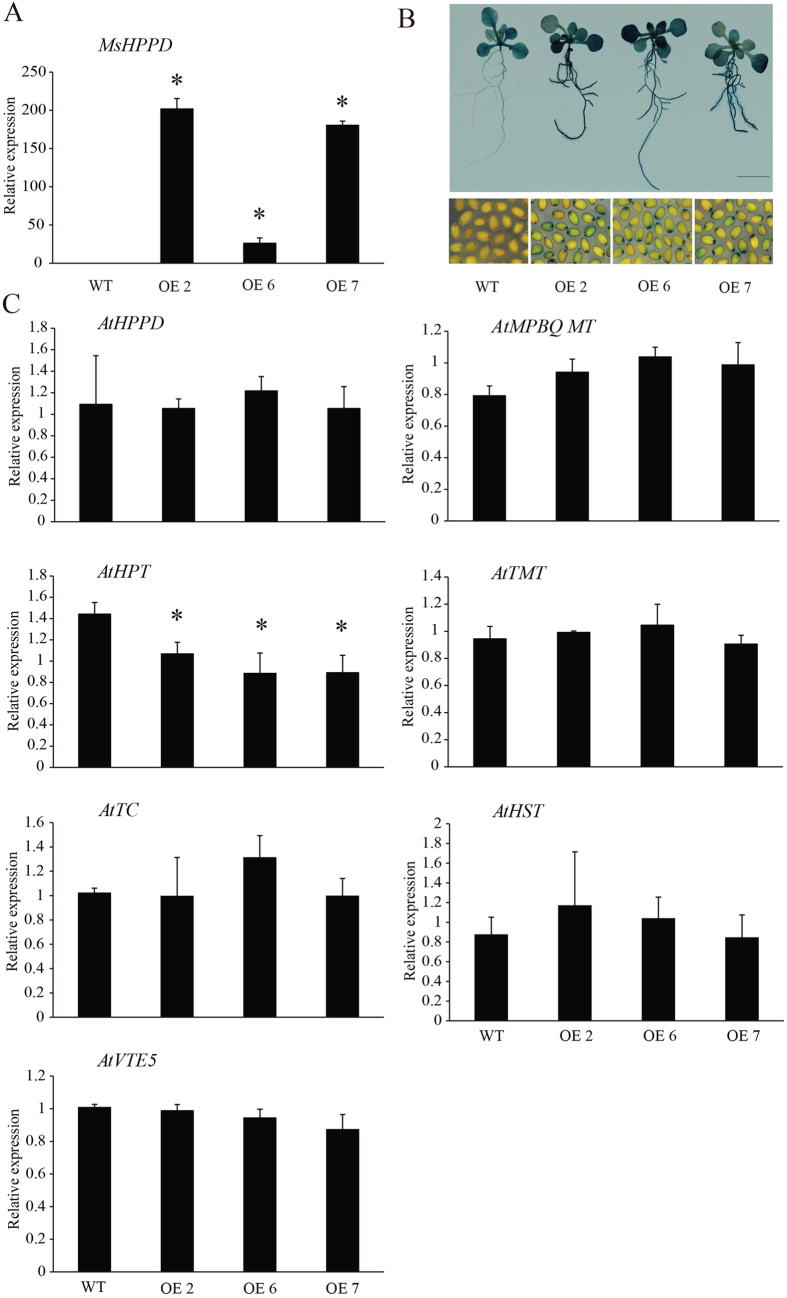
(**A**) *MsHPPD* expression in three *MsHPPD-*overexpressing transgenic lines and wild-type, as measured by qRT-PCR. Total RNA was extracted from two-week-old *MsHPPD-*overexpressing transgenic *Arabidopsis* (OE2, OE6, and OE7) and control seedlings, before being subjected to qRT-PCR analysis. The expression levels of *MsHPPD* were normalized to *At4g26410*. Data are presented as mean ± SD, each with three biological replicates and three technical replicates. Statistical analyses were carried out via a two-tailed Student’s *t-*test with a significance of P < 0.05. (**B**) Histochemical GUS staining of *MsHPPD-*overexpressing lines (OE2, OE6, and OE7) and wild-type seedlings with pBI121 empty vector in the Col-0 background. Two-week-old transgenic and control *Arabidopsis* were used for GUS staining. Bar: 1 cm. (**C**) Expression analysis of endogenous genes involved in vitamin E biosynthesis in *MsHPPD-* overexpressing *Arabidopsis* (OE2, OE6, and OE7) and wild-type control. Two-week-old *Arabidopsis* seedlings were used for qRT-PCR. Data are shown as the mean ± SD with three biological replicates and three technical replicates. The expression levels of the genes detected in the experiment were normalized to *At4g26410*. Statistical analyses were carried out via a two-tailed Student’s *t* tests with a significance of P < 0.05.

qRT-PCR was used to examine the expression levels of *MsHPPD* in *MsHPPD-*overexpressing lines and wild-type *Arabidopsis*. This data showed that *MsHPPD* was more-highly expressed in transgenic *Arabidopsis* lines, to varying degrees, as compared to wild-type expression levels (Fig. 4A). Likewise, all of the pHPPD::GUS transgenic *Arabidopsis* lines exhibited higher levels of GUS activity compared with the corresponding wild-type plants (Fig. 4B), which was consistent with the transcriptional levels of *MsHPPD*. To test whether the overexpression of exogenous *MsHPPD* alters the endogenous vitamin E biosynthetic pathway, the expression levels of critical endogenous vitamin E pathway genes were quantified. There are several genes that have been shown to regulate vitamin E biosynthesis. *HPPD* and *HOMOGENTISATE PHYTYLRANSFERASE (HPT*) have been found to play roles pertaining to determining the total vitamin E content, while *2-METHYL-6-PHYTYLBENZOQUINONEMETHYLTRANSFERASE (MPBQMT*) and *γ-TOCOPHEROL METHYL TRANSFERASE (γ-TMT)* play roles in determining the vitamin E composition^18^. A significant decrease of *AtHPT*, which plays a role in determining vitamin E content and is also the first gene downstream of *HPPD* in vitamin E biosynthesis, was observed in the generated *MsHPPD*-overexpressing lines, but the expression levels of *AtMPBQMT, AtTMT, AtTC, AtHPPD and AtVTE5* were not in the wild-type. Since HPPD is also involved in plastoquinone-9 and plastochromanol-8 biosynthesis, the expression level of *AtHST*, which is the first gene downstream of HPPD in plastoquinone-9 and plastochromanol-8 biosynthesis, was examined, and no significant difference was observed. This suggests that the plastoquinone-9 and plastochromanol-8 biosynthetic pathway is not altered (Fig. 4C).

### Analysis of vitamin E levels in transgenic *Arabidopsis* seeds and leaves

**Figure 5:**
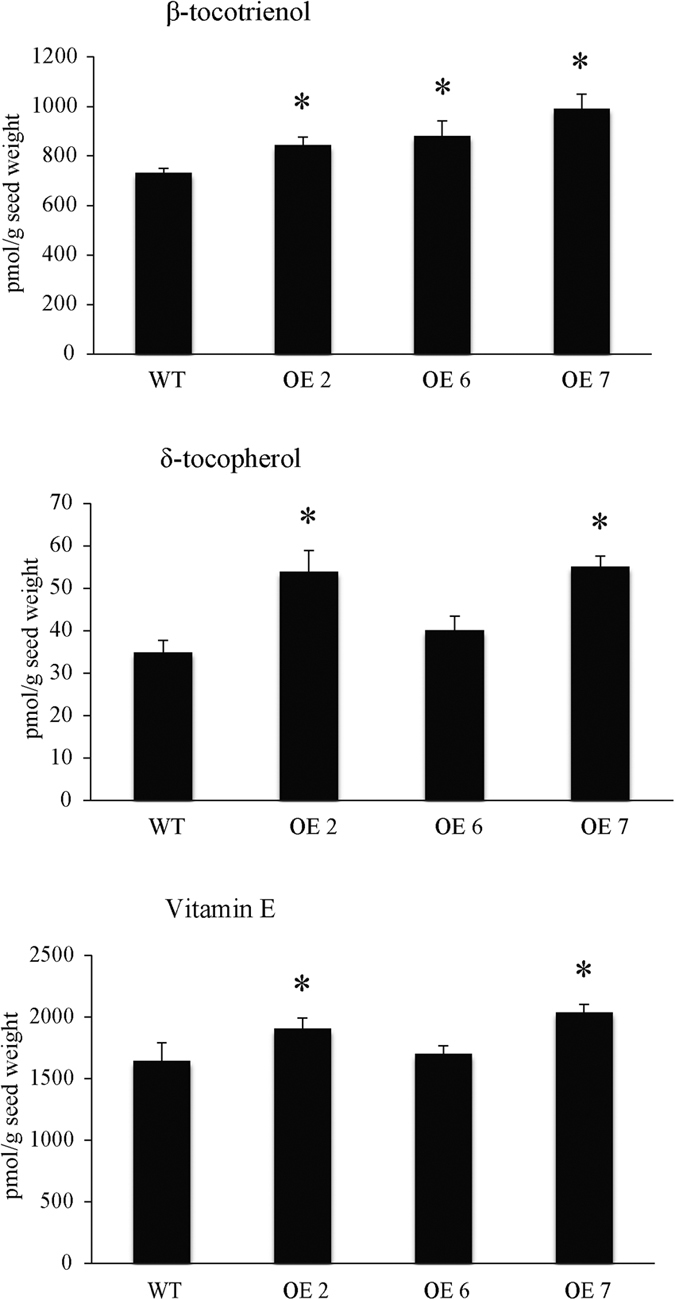
Analyses of vitamin E component in transgenic *Arabidopsis* seeds. Seeds from *MsHPPD-*overexpressing lines (OE2, OE6 and OE7) and wild-type *Arabidopsis* were collected at the same time, air dried for two weeks and used for this measurement. Data are presented as mean ± SD with four biological replicates. Statistical analyses were carried out via a two-tailed Student’s *t-*test with a significance of P < 0.05.

To test whether *MsHPPD* is a functional gene involved in the production of tocotrienols and tocopherols, all vitamin E forms were quantified in the seeds and leaves of *MsHPPD-*overexpressing lines and wild-type *Arabidopsis*. β-tocotrienol and δ-tocopherol content were found to have increased by 12–36% and 10–57%, respectively, in transgenic *Arabidopsis* seeds compared to wild-type seeds (Fig. 5). However, δ-tocotrienol was undetectable in all lines and the content of the other forms of vitamin E was not altered (Fig. S2A). Total vitamin E content was significantly higher in the seeds of *MsHPPD-*overexpressing lines OE2 and OE7 than the corresponding content in the seeds of the control line (Fig. 5). This corresponds to the previously obtained *MsHPPD* expression levels, in which lines OE2 and OE7 had the most significantly enhanced *MsHPPD* expression, compared with OE6 and wild-type (Fig. 4A). In *Arabidopsis* leaves, δ-tocopherol, γ-tocopherol, β-tocotrienol and γ-tocotrienol were not detected, and no significant difference was ultimately observed with regards to total vitamin E content or the levels of each quantifiable vitamin E form (Fig. S2B).

### Effect of *MsHPPD* on seed germination in *Arabidopsis*

**Figure 6:**
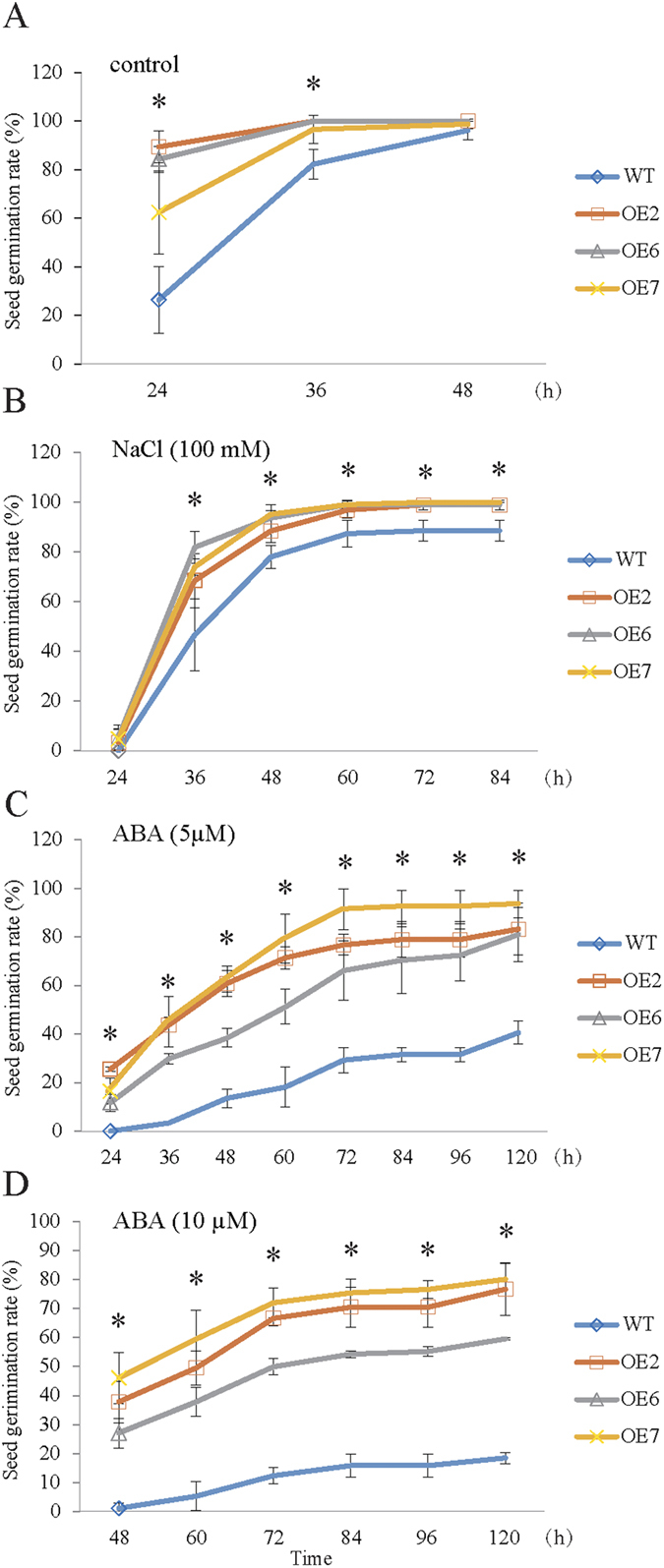
Effect of *MsHPPD* on seed germination in transgenic *Arabidopsis*. (**A**) Seed germination rate under normal conditions. (**B**) Seed germination rate under NaCl treatment. (**C** and **D**) Seed germination rate under ABA treatment. Seeds of transgenic and control *Arabidopsis* were harvested at the same time, dried at room temperature for two weeks, and germinated on half-strength MS medium, either with or without the applicable treatments. Seed germination rate was calculated based on four biological replicates (~80 seeds per replicate). Data are presented as mean ± SD. Statistical analyses were carried out via a two-tailed Student’s *t-*test with a significance of P < 0.05.

To explore the potential role of the increased β-tocotrienol in transgenic *Arabidopsis* seeds, seed germination rate was examined under standard conditions, as well as under various stress conditions. Under normal conditions, the seed germination rate of *MsHPPD-*overexpressing transgenic lines was significantly higher than that of wild-type at 24 h. However, as the time after imbibition increased, the difference between the germination rates of transgenic and wild-type lines diminished, and so the difference in germination rate was less pronounced after 36 h, and by 48 h, both wild-type and transgenic lines reached equivalent germination rates under standard growth conditions (Fig. 6A). Under NaCl treatment, the seed germination rate of both transgenic and wild-type *Arabidopsis* lines was inhibited, however, the seed germination rate was significantly higher in the seeds of all transgenic lines compared to the corresponding rate observed in wild-type seeds, and this trend holds up at all tested time points. Ultimately, after 84 h, the germination rate of both the transgenic and control lines was determined to be 99% and 88%, respectively (Fig. 6B). Under 5 μM ABA treatment, the seed germination rate of *MsHPPD-*overexpressing transgenic lines at 120 h was approximately 86%, and this was significantly higher than the germination rate of the control line, which was found to be 40% (Fig. 6C). Furthermore, when the ABA concentration was doubled to 10 μM, the seed germination rate of the transgenic lines at 120 h decreased slightly to around 72%, but the germination rate of the wild-type lines decreased much more drastically to 18% (Fig. 6D).

### Expression of genes involved in the ABA biosynthetic and signaling and total ABA content are significantly reduced in seeds of *MsHPPD-*overexpressing *Arabidopsis*

**Figure 7:**
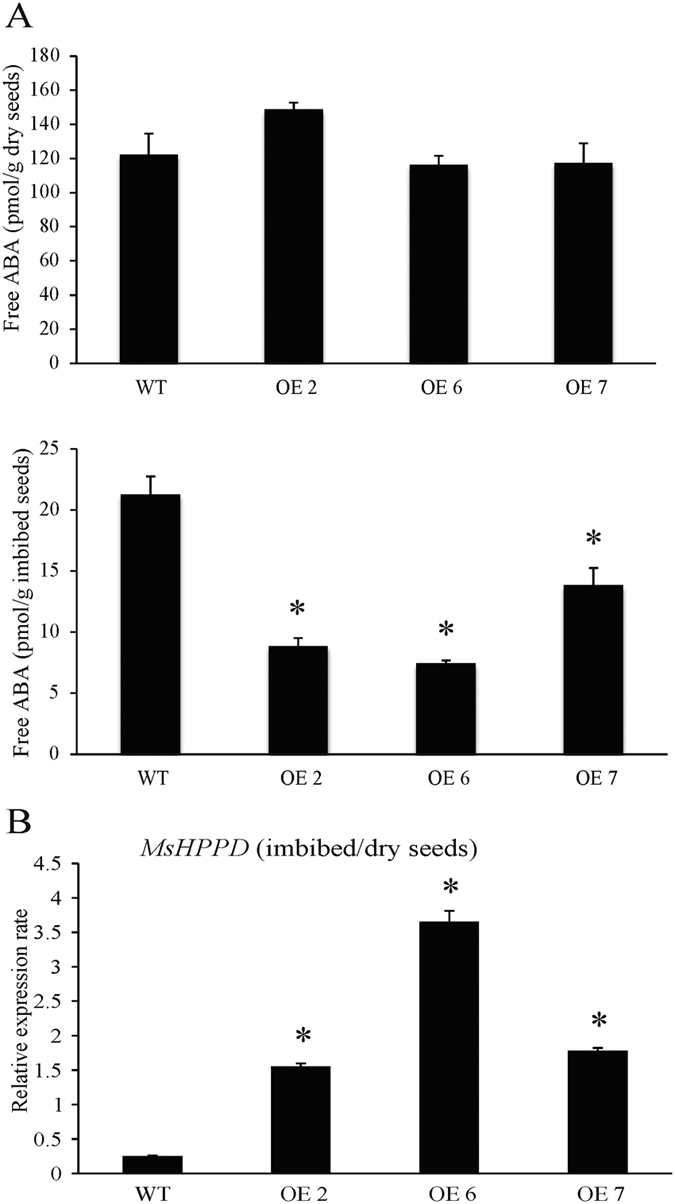
Effects of *MsHPPD* on ABA content in transgenic *Arabidopsis*. (**A**) ABA content in dry and imbibed *MsHPPD-*overexpressing *Arabidopsis* (OE2, OE6, and OE7) and wild-type seeds. Transgenic and control *Arabidopsis* seeds were harvested at the same time, dried at room temperature for two weeks, and used for ABA measurement in dry seeds (upper plot). Alternatively, they were submerged in water for 36 h and used for ABA measurement in imbibed seeds (lower plot). Data are presented as mean ± SD using three biological replicates. Statistical analyses were carried out via a two-tailed Student’s *t-*test with a significance of P < 0.05. (**B**) Expression rate of *MsHPPD* in imbibed seeds relative to dry seeds of *MsHPPD*-overexpressing transgenic and control *Arabidopsis*. The samples used here were the same as those in A. Data are presented as mean ± SD using three biological replicates and three technical replicates. Statistical analyses were carried out via a two-tailed Student’s *t-*test with a significance of P < 0.05.

**Figure 8:**
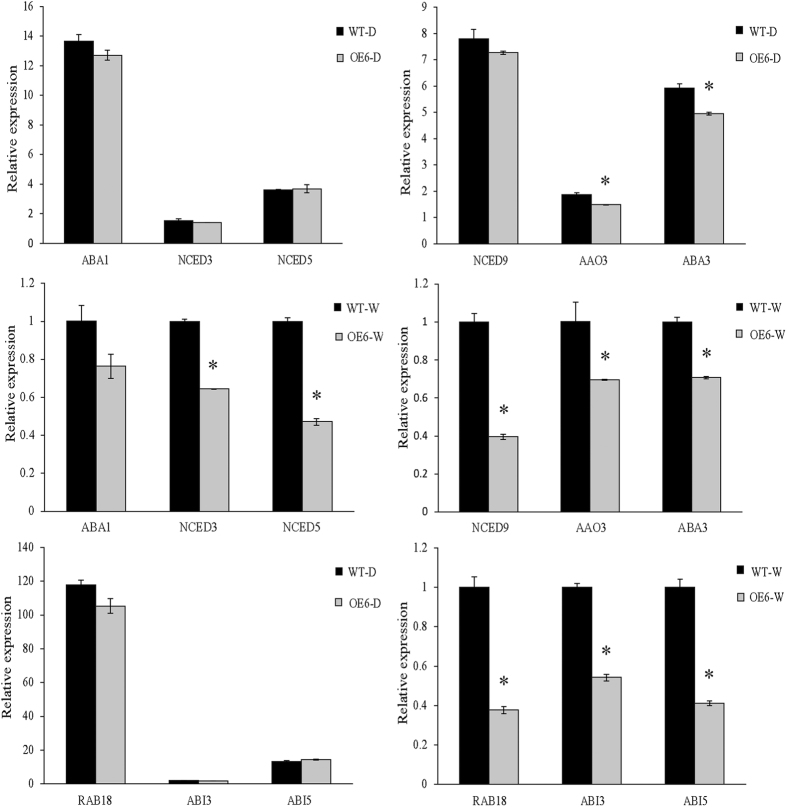
Expression levels of ABA biosynthesis and ABA signaling pathway genes in *MsHPPD-*overexpressing transgenic *Arabidopsis*. The samples used here were the same as those in Fig. 7. Data are presented as mean ± SD using three biological replicates and three technical replicates. The expression levels of the genes detected in the experiment were normalized to *At4g26410*. Statistical analyses were carried out via a two-tailed Student’s *t-*test with a significance of P < 0.05.

Since seed germination rate was significantly affected in the *MsHPPD-*overexpressing lines and ABA is an important phytohormone involved in seed dormancy and germination, we quantified the free ABA level in dry seeds from *MsHPPD-*overexpressing lines and wild-type. In addition, since a significant difference was observed in the seed germination rate between *MsHPPD-*overexpressing lines and wild-type imbibed seeds at 36 h, the ABA level in imbibed seeds were also measured. While no significant difference was observed in dry seeds, ABA content was significantly decreased in imbibed *MsHPPD-*overexpressing *Arabidopsis* seeds compared to imbibed wild-type seeds (Fig. 7A). Meanwhile, the expression levels of *MsHPPD* in *MsHPPD-*overexpressing lines were significantly higher in imbibed seeds than they were in dry seeds (Fig. 7B). Among the three *MsHPPD-*overexpressing lines, OE6 showed the dramatically highest *MsHPPD* expression level when imbibed with water and significantly reduced ABA content providing us with a suitable transgenic line to evaluate the relationship between *MsHPPD* expression level and ABA content. To determine whether the decrease of ABA levels in transgenic lines was due to changes in ABA biosynthesis or in ABA metabolism, qRT-PCR was performed to examine the expression levels of the ABA biosynthetic and signaling pathway genes in OE6. The results showed that the expression of the ABA biosynthetic genes *ABA1, NCED3, NCED5* and *NCED9* was not altered in dry seeds of the *MsHPPD-*overexpressing lines; however, in imbibed seeds, *NCED3, NCED5* and *NCED9* expression was significantly reduced, compared to the expression in wild-type seeds. Meanwhile, the expression levels of two other ABA biosynthetic genes, *AAO3* and *ABA3,* were both significantly lower in the *MsHPPD-*overexpression lines than they were in wild-type seeds, regardless of whether the seeds were dried or imbibed. Likewise, the expression levels of the ABA signaling pathway genes *RAB18, ABI3,* and *ABI5* were not changed in dry seeds, but were significantly down-regulated in imbibed seeds of the *MsHPPD-*overexpressing lines compared to wild-type seeds (Fig. 8).

## Discussion

HPPD catalyzes the first committed step in vitamin E biosynthesis^2^. Although it has been studied in many organisms^27^,^28^,^29^,^30^; the function of the *HPPD* gene in the economically important forage plant alfalfa remains unknown. In this study, full-length *HPPD* cDNA from alfalfa was isolated, and subsequent amino acid sequence alignment showed that MsHPPD from alfalfa shared high similarity with *LsHPPD* from *Lactuca sativa, MtHPPD* from *Medicago truncatula,* and *AtHPPD* from *Arabidopsis thaliana*. Among these HPPDs, *LsHPPD* and *AtHPPD* have been previously implicated as being able to alter vitamin E content in transgenic plants^29^,^31^. Phylogenetic tree construction also revealed that *MsHPPD* was evolutionally closest to HPPD sequences from these dicotyledonous plants.

Expression pattern analyses showed that NaCl and PEG treatment rapidly and robustly induced *MsHPPD* expression, suggesting that *MsHPPD* is involved in multiple stress responses in alfalfa. Darkness was also found to induce the expression of *MsHPPD*, and this is in agreement with the results from Falk *et al*.^32^, who found that *HPPD* expression levels increased during dark-induced leaf senescence. This may be due to the breakdown of chlorophyll-released free phytol, a precursor for tocopherol synthesis, and thus increased the demand for *HPPD*^33^,^34^. When plants were transferred from darkness to light, *MsHPPD* expression dropped sharply and continued to decrease (Fig. 3D), which suggests that light acts as a negative regulator of *MsHPPD* expression.

ABA induced the expression of *MsHPPD* modestly, albeit significantly. *MsHPPD* expression levels were initially high in primary roots and cotyledons, and increased significantly after the ABA induction. It has been shown that salicylic acid (SA), methyl jasmonic acid (MeJA), and high light can induce the expression of both *SmHPPD* from *Salvia miltiorrhiza* and also *AtHPPD*^30^,^35^. MeJA treatment was not found to alter the expression levels of *MsHPPD*, which might be a manifestation of the lack of a MeJA-responsive element in the promoter region of *MsHPPD* (Fig. S1). However, in agreement with the previous study, SA has significantly induced the expression of *MsHPPD*. Taken together, our results suggest that *HPPD* could be induced by multiple conditions, and the flexible expression levels of *MsHPPD* in response to stresses implied that *MsHPPD* plays multiple important roles with regards to responding to stresses.

In the leaves of *MsHPPD-*overexpressing transgenic lines, no difference was recorded to either vitamin E composition or vitamin E content (Fig. S2). This was contradictory to the result obtained by Tsegaye *et al*.^31^, who reported that overexpressing *AtHPPD* increased tocopherol by around 37% in transgenic *Arabidopsis* leaves. However, overexpressing barley *HPPD* had no effect on the tocopherol content in transgenic tobacco leaves^11^. This discrepancy may be attributable to subtle differences between the different organisms used in these studies. To gain a deeper insight into the reason for this discrepancy, we tested the expression levels of endogenous vitamin E biosynthetic genes in *Arabidopsis* to examine whether *MsHPPD* overexpression affected the flux of the entire vitamin E biosynthetic pathway. Interestingly, we observed a significant decrease only in the expression level of endogenous *AtHPT* in transgenic lines, suggesting that the vitamin E biosynthesis pathway is tightly controlled. The increased expression levels of *MsHPPD* appear to have somehow inhibited the expression of *AtHPT*, which also plays a role in determining vitamin E accumulation along with *HPPD*, and the inhibition of *AtHPT* may counteract the effects caused by the enhancement of *MsHPPD* expression on vitamin E to an unknown degree. As such, perhaps this explains why no significant differences were observed regarding the vitamin E content of the leaves.

As was previously reported, the content and composition of vitamin E varies in different tissues and different developmental stages^8^. α-tocopherol is the dominant form found in plant leaves, while γ-tocopherol and tocotrienols mainly exist in seeds^36^. This phenomenon made us question the function of elevated β-tocotrienol in seeds; therefore, we tested the seed germination rate of seeds from *MsHPPD-*overexpressing *Arabidopsis* lines and also wild-type seeds, under both normal and stressful conditions. Under normal conditions, the transgenic lines germinated earlier than wild-type plants. Meanwhile, under NaCl treatment, the germination of both transgenic and wild type lines was inhibited compared to normal conditions; however, the germination rate of the seeds from transgenic lines was significantly higher than that of wild type seeds. These results suggest that *MsHPPD* plays a positive role in regulating seed germination.

Seed eFP browser^37^ showed that *HPPD* belongs to the ABA group of genes and that it might be involved in ABA biosynthesis or the ABA signaling pathway. Here, we showed that transgenic *Arabidopsis* lines which overexpressing *MsHPPD* were less sensitive to ABA treatment, in terms of seed germination rate (Fig. 6C,D). Additionally, the ABA content was significantly lower in imbibed transgenic *Arabidopsis* seeds than it was in imbibed wild-type seeds. However, the expression level of *MsHPPD* was significantly higher in imbibed transgenic *Arabidopsis* seeds. Since vitamin E and ABA are both downstream of GGDP, overexpression of *MsHPPD* could increase in vitamin E content, thus decreasing the amount of GGDP substrate available for ABA biosynthesis. Expression of the ABA biosynthetic genes *NCED3, NCED5* and *NCED9* was found to be unaltered in dry seeds; but was significantly reduced in imbibed transgenic *Arabidopsis* seeds (Fig. 8), suggesting that the reduced GGDP pool size decreased the expression level of ABA biosynthetic genes. In addition, expression of the ABA signaling pathway genes *ABI3* and *ABI5*, which are important for seed germination, decreased significantly in imbibed transgenic *Arabidopsis* seeds. Based on the above data, it is possible that vitamin E and ABA compete for GGDP, and, thus, elevated vitamin E necessitate reduced ABA levels, and indeed, this is what we observed (Fig. S3). In turn, the reduced ABA content led to a weakening of ABA signaling, ultimately resulting in either a faster-than-normal or higher-than-normal germination rate in the seeds of the transgenic *MsHPPD-*overexpressing lines, compared with the germination rate of the corresponding control, under various conditions. Moreover, vitamin E has been demonstrated to have additional biological activities besides its antioxidant functions in mammalian systems. Specific tocopherols have been found to modulate signal transduction pathways^38^,^39^. For instance, tocopherol-binding proteins can regulate gene transcriptional activities^40^. Although a regulatory activity for tocopherols has not yet been directly proven in land plants, the present data raises the possibility that vitamin E also modulates signal transduction pathways in plants. Specifically, tocopherol may modulate seed germination and other hormone responses by binding with other proteins to regulate the ABA signaling pathway. Additional work is necessary for these hypotheses to be investigated in future studies.

In conclusion, an *HPPD* gene that encodes a 434 amino acid protein, named *MsHPPD*, was isolated from alfalfa. *MsHPPD* was shown to be induced by multiple treatments, and overexpression of *MHPPD* in *Arabidopsis* increased the total vitamin E content. Seed germination of the transgenic lines was accelerated under normal conditions, and was less strongly inhibited under stress treatments. Overexpression of *MsHPPD* was also found to significantly reduce the free ABA content, as well as the expression of ABA biosynthetic genes and ABA signaling pathway genes. This represents the first evidence that vitamin E is involved in regulating germination rate via affecting the ABA biosynthesis and the subsequent ABA signaling pathway.

## Methods

### Plant materials and growth conditions

*Arabidopsis* (Columbia-0) was used for these present experiments. Seeds were surface sterilized with 70% ethanol for 2 min and 5% sodium hypochlorite for 5 min, and then washed three times with water. The sterilized seeds were planted on half-strength MS medium (2.2 g/L Murashige and Skoog salts, pH 5.7, and 8 g/L agar), and kept at 4 °C for 2 days to stratify the seeds and facilitate seed germination. The *Arabidopsis* plants were grown under long-day photoperiod conditions (16 h light/8 h dark at 25 °C). Seeds from *Medicago sativa* L. cv Zhongmu No. 1 were germinated on wet filter paper, and the seedlings were grown under long-day photoperiod conditions as above, after the appearance of the first true leaf.

### Gene cloning, vector construction and transformation

The full-length *MsHPPD* gene was cloned from cDNA prepared from alfalfa leaves via rapid amplification of cDNA ends (RACE), according to the manufacturer’s protocol (Clontech, USA). The promoter of *MsHPPD* was cloned via chromosome walking. The binary vector pBI121^41^ with a GUS reporter gene was used to express *MsHPPD* under the control of the constitutive CaMV 35 S promoter, and the CDS of *MsHPPD* without a stop codon was introduced into PBI121 and fused with the GUS reporter gene. For the gene expression analysis, the CaMV 35 S promoter was replaced with the native promoter of *MsHPPD,* which was fused to the GUS reporter gene. The constructs harboring *MsHPPD* and *pMsHPPD* were transformed into the agrobacterial strain GV3101, which was then used for *Arabidopsis* transformation via the well-established floral dipping method^42^.

### Treatments

Three-week-old Zhongmu No. 1 seedlings, which were grown in half-strength MS nutrient water, were subjected to different treatments. NaCl, ABA, PEG, SA, and MeJA treatments were performed by adding the compounds to the nutrient water and collecting leaf tissue at 0, 2, 4, 8, 12 and 24 h after treatment. The final concentrations of NaCl, ABA, PEG, SA, and MeJA were 100 mM, 0.1 mM, 300 mM, 0.1 mM, and 0.1 mM, respectively^43^. Light treatment was performed by growing seedlings in dark conditions, and then collecting leaf tissue at 0, 12, 24, 48, and 72 h, and a second group of experiments was performed by exposing 72 h dark-treated seedlings to light and collecting leaf tissue at 0.25, 0.5, 6, and 12 h.

### Quantitative real-time PCR

Total RNA was isolated using TRIzol (Invitrogen, USA) and treated with DNase I to eliminate DNA contamination. One microgram of total RNA was then used for cDNA synthesis using the SuperScript III First-Strand Synthesis System (Invitrogen, USA). qRT-PCR was performed with an ABI 7500 Real-Time PCR System (Bio-Rad, USA) using the SYBR Green PCR Master Mix (Takara, Japan) and the appropriate primers (Table 1). The relative gene expression levels were calculated by 2^−ΔΔCt ^44. *At4g26410*^45^ and *Actin*^25^ were used as reference genes to normalize the expression levels of the target genes in *Arabidopsis* and alfalfa, respectively. All experiments were performed using three biological replicates and three technical replicates.

### GUS histochemical assay

A GUS histochemical assay was performed according to the method described by Jefferson *et al*.^46^. Plant tissues were incubated at 37 °C overnight in 50 mM sodium pyrophosphate staining buffer (pH 7.2) containing 2 mM X-Gluc (5-bromo-4-chloro-3-indolyl β-D-glucuronide), 10 mM EDTA, 2 mM potassium ferricyanide, 2 mM potassium ferrocyanide, and 0.2% (v/v) Triton X-100. 100% ethanol was used to completely remove chlorophyll from tissues.

### Extraction and HPLC analyses of tocopherols and tocotrienols

Extractions and analyses of tocopherols and tocotrienols were performed according to the method described before^25^.

### Seed germination

*MsHPPD-*overexpressing and wild-type *Arabidopsis* seeds were surface-sterilized as described above and planted on half-strength MS medium with the presence or absence of 100 mM NaCl, 5 μM ABA, or 10 μM ABA. After being stratified at 4 °C for 3 days, the seeds were transferred to a growth chamber (16 h light/8 h dark at 25 °C) and germination rate was monitored every 12 hours.

### ABA measurement in *Arabidopsis* seeds

For each replicate, approximately 20 mg (dry weight) of seeds were homogenized under liquid nitrogen, weighed, and extracted for 24 h in cold methanol and 2H6-ABA (internal standard, OlChemIm, Czech Republic). Endogenous ABA was purified and measured as previously described^47^ with some modifications to the detection conditions. LC-MS/MS analysis was performed on a UPLC system (Waters) coupled to the 5500 QTRAP system (AB SCIEX, USA), and a BEH C18 column (1.7 mm, 2.1 × 150 mm; Waters) was used for LC separation with mobile phase 0.05% HAc (A) and 0.05% HAc in ACN (B). The gradient was set with initial 20% B and increased to 70% B within 6 min. ABA was detected in MRM mode with transition 263/153. Finally, quantitation was performed using the isotope dilution method.

### Statistical analysis

Statistical analyses were performed by two-tailed Student’s *t*-test. The difference was considered significant at P < 0.05. Data presented are mean ± SD.

## Additional Information

**How to cite this article**: Jiang, J. *et al*. *P-HYDROXYPHENYLPYRUVATE DIOXYGENASE* from *Medicago sativa* is involved in vitamin E biosynthesis and abscisic acid-mediated seed germination. *Sci. Rep.* **7**, 40625; doi: 10.1038/srep40625 (2017).

**Publisher's note:** Springer Nature remains neutral with regard to jurisdictional claims in published maps and institutional affiliations.

## Supplementary Material

Supplementary Information

## Figures and Tables

**Table 1:** The primer sequences used in cloning and expression analysis.

Primers	Sequences	Application
3′RACE	GTGCCATTCCATCGTCTCCTCCA	RACE
5′RACE	GGATTCTGGTTTGGGTTCGGGTC	RACE
PBI-HPPD-F	CACGGGGGACTCTAGAATGGCCATCGAAACAGAAAC	Vector construction
PBI-HPPD-R	AGGGACTGACCACCCGGGGATGCAGTTCTTCTAGTTTCCA	Vector construction
AtHPPD-F	GCGGTTTAAATTCAGCGGTCCTG	Q-PCR
AtHPPD-R	TCCGTGCACTGGCTCGTTAATC	Q-PCR
AtHPT-F	TGGAGCAAAGTCATCTCGGTTG	Q-PCR
AtHPT-R	GACTTAGCTCGAGCCCACAAAG	Q-PCR
AtTC-F	TGTCCGAAGGGTTCCAAGCTAC	Q-PCR
AtTC-R	CACAGTTTCCGCATAGTCAGTACG	Q-PCR
AtMPBQMT-F	TGCCGGTTTCAAGGACGTTCAG	Q-PCR
AtMPBQMT-R	TCTTCCTTTGGACCAAGCTGGAG	Q-PCR
AtTMT-F	TGCTCAATCACTCGCTCATAAGGC	Q-PCR
AtTMT-R	TCTTCGAATGGCTGATCCAACGC	Q-PCR
VTE5-F	CAAGCGAAACGTCATTCAACAGAG	Q-PCR
VTE5-R	GAAGATTGGCCACGCAAGTACG	Q-PCR
AtHST-F	GCATGTTCTCAGGTTGGTGCTG	Q-PCR
AtHST-R	AGCTCTTGTCACCAAGGCAGTG	Q-PCR
AtABI3-F	GAAGCAAAGCGACGTGGGTAAC	Q-PCR
AtABI3-R	AACCTGTAGCGCATGTTCCAAAC	Q-PCR
AtABI5-F	AAACATGCATTGGCGGAGTTGG	Q-PCR
AtABI5-R	CGGTTGTGCCCTTGACTTCAAAC	Q-PCR
AtABA1-F	TGGGCTTGGTCCTCTGTCTTTC	Q-PCR
AtABA1-R	CACCAACTCTTCCTGGATGTGG	Q-PCR
AtRAB18-F	TCTAGCTCGGAGGATGATGGAC	Q-PCR
AtRAB18-R	ACCGTAGCCACCAGCATCATATC	Q-PCR
AtNCED3-F	TCGAAGCAGGGATGGTCAACAG	Q-PCR
AtNCED3-R	GCTCGGCTAAAGCCAAGTAAGC	Q-PCR
AtNCED5-F	CCTCCGTTAGTTTCACCAACACT	Q-PCR
AtNCED5-R	GGTGTGTCGGAGACGGAGTT	Q-PCR
AtNCED9-F	CCCAAACCGCAGCGTTTAAT	Q-PCR
AtNCED9-R	GTTCGGTCGAGGAATTGGGT	Q-PCR
AtABA3-F	TGCTACAAGGCTCCCCCTTT	Q-PCR
AtABA3-R	CACAACCCTTTGCAGCATCA	Q-PCR
AtAAO3-F	GAAGGTCTTGGAAACACGAAGAA	Q-PCR
AtAAO3-R	GAAATACACATCCCTGGTGTACAAAAC	Q-PCR
MsHPPD-F	CAGTTATCCTCGAAAACCACG	Q-PCR
MsHPPD-R	GTTTGGGTTCGGGTCGTTG	Q-PCR
Actin-F	GAGCGTTTCCGTTGTCCTGA	Q-PCR
Actin-R	AGGTGCTGAGGGAAGCCAAA	Q-PCR

## References

[b1] Miret J. A. & Munne-Bosch S. Redox signaling and stress tolerance in plants: a focus on vitamin E. Ann N Y Acad Sci. 1340, 29–38 (2015). 25586886 10.1111/nyas.12639

[b2] Abbasi A. R., Hajirezaei M., Hofius D., Sonnewald U. & Voll L. M. Specific roles of alpha- and gamma-tocopherol in abiotic stress responses of transgenic tobacco. Plant Physiol. 143, 1720–1738 (2007). 17293434 10.1104/pp.106.094771 PMC1851823

[b3] Dal Bosco A., Mattioli S., Ruggeri S., Mugnai C. & Castellini C. Effect of slaughtering age in different commercial chicken genotypes reared according to the organic system: 2. Fatty acid and oxidative status of meat. Ital J of Anim Sci. 13 (2014).

[b4] Traber M. G. Vitamin E inadequacy in humans: causes and consequences. Adv Nutr. 5, 503–514 (2014). 25469382 10.3945/an.114.006254 PMC4188222

[b5] Evans H. M. & Bishop K. S. On the Existence of a Hitherto Unrecognized Dietary Factor Essential for Reproduction. Science. 56, 650–651 (1922). 17838496 10.1126/science.56.1458.650

[b6] Sattler S. E. Characterization of Tocopherol Cyclases from Higher Plants and Cyanobacteria. Evolutionary Implications for Tocopherol Synthesis and Function. Plant Physiol. 132, 2184–2195 (2003). 12913173 10.1104/pp.103.024257 PMC181302

[b7] Hofius D. & Sonnewald U. Vitamin E biosynthesis: biochemistry meets cell biology. Trends Plant Sci. 8, 6–8 (2003). 12523993 10.1016/s1360-1385(02)00002-x

[b8] DellaPenna D. A decade of progress in understanding vitamin E synthesis in plants. J Plant Physiol. 162, 729–737 (2005). 16008096 10.1016/j.jplph.2005.04.004

[b9] Sakuragi Y., Maeda H., Dellapenna D. & Bryant D. A. Alpha-Tocopherol plays a role in photosynthesis and macronutrient homeostasis of the cyanobacterium Synechocystis sp. PCC 6803 that is independent of its antioxidant function. Plant Physiol. 141, 508–521 (2006). 16565298 10.1104/pp.105.074765 PMC1475434

[b10] Norris S. R., Barrette T. R. & DellaPenna D. Genetic dissection of carotenoid synthesis in arabidopsis defines plastoquinone as an essential component of phytoene desaturation. Plant Cell. 7, 2139–2149 (1995). 8718624 10.1105/tpc.7.12.2139 PMC161068

[b11] Falk J., Andersen G., Kernebeck B. & Krupinska K. Constitutive overexpression of barley 4-hydroxyphenylpyruvate dioxygenase in tobacco results in elevation of the vitamin E content in seeds but not in leaves. FEBS Letters. 540, 35–40 (2003). 12681479 10.1016/s0014-5793(03)00166-2

[b12] Karunanandaa B. . Metabolically engineered oilseed crops with enhanced seed tocopherol. Metab Eng. 7, 384–400 (2005). 16125431 10.1016/j.ymben.2005.05.005

[b13] Crowell E. F., McGrath J. M. & Douches D. S. Accumulation of vitamin E in potato (Solanum tuberosum) tubers. Transgenic Res. 17, 205–217 (2008). 17415670 10.1007/s11248-007-9091-1

[b14] Seiler C. . ABA biosynthesis and degradation contributing to ABA homeostasis during barley seed development under control and terminal drought-stress conditions. J Exp Bot. 62, 2615–2632 (2011). 21289079 10.1093/jxb/erq446

[b15] Guajardo E., Correa J. A. & Contreras-Porcia L. Role of abscisic acid (ABA) in activating antioxidant tolerance responses to desiccation stress in intertidal seaweed species. Planta. 243, 767–781 (2015). 26687373 10.1007/s00425-015-2438-6

[b16] Nambara E. & Marion-Poll A. Abscisic acid biosynthesis and catabolism. Annu Rev Plant Biol. 56, 165–185 (2005). 15862093 10.1146/annurev.arplant.56.032604.144046

[b17] Hirai N., Yoshida R., Todoroki Y. & Ohigashi H. Biosynthesis of abscisic acid by the non-mevalonate pathway in plants, and by the mevalonate pathway in fungi. Biosci Biotechnol Biochem. 64, 1448–1458 (2000). 10945263 10.1271/bbb.64.1448

[b18] DellaPenna D. & Pogson B. J. Vitamin synthesis in plants: tocopherols and carotenoids. Annu Rev Plant Biol. 57, 711–738 (2006). 16669779 10.1146/annurev.arplant.56.032604.144301

[b19] Lefebvre V. . Functional analysis of Arabidopsis NCED6 and NCED9 genes indicates that ABA synthesized in the endosperm is involved in the induction of seed dormancy. Plant J. 45, 309–319 (2006). 16412079 10.1111/j.1365-313X.2005.02622.x

[b20] Kim H. . ABA-HYPERSENSITIVE BTB/POZ PROTEIN 1 functions as a negative regulator in ABA-mediated inhibition of germination in Arabidopsis. Plant Mol. Biol. 90, 303–315 (2016). 26667153 10.1007/s11103-015-0418-7

[b21] Komatsu K. . Group A PP2Cs evolved in land plants as key regulators of intrinsic desiccation tolerance. Nat Commun. 4, 2219 (2013). 23900426 10.1038/ncomms3219 PMC3731658

[b22] O’Rourke J. A. . The Medicago sativa gene index 1.2: a web-accessible gene expression atlas for investigating expression differences between Medicago sativa subspecies. BMC Genomics. 16, 502 (2015). 26149169 10.1186/s12864-015-1718-7 PMC4492073

[b23] Long R. . Isolation and functional characterization of salt-stress induced RCI2-like genes from Medicago sativa and Medicago truncatula. J Plant Res. 128, 697–707 (2015). 25801273 10.1007/s10265-015-0715-x

[b24] Armstrong J. M. CYTOLOGICAL STUDIES IN ALFALFA POLYPLOIDS. Canadian Journal of Botany. 32, 531–542 (1954).

[b25] Jiang J. . Overexpression of Medicago sativa TMT elevates the alpha-tocopherol content in Arabidopsis seeds, alfalfa leaves, and delays dark-induced leaf senescence. Plant Sci. 249, 93–104 (2016). 27297993 10.1016/j.plantsci.2016.05.004

[b26] Lescot M. . PlantCARE, a database of plant cis-acting regulatory elements and a portal to tools for in silico analysis of promoter sequences. Nucleic Acids Res. 30, 325–327 (2002). 11752327 10.1093/nar/30.1.325 PMC99092

[b27] Matringe M., Ksas B., Rey P. & Havaux M. Tocotrienols, the unsaturated forms of vitamin E, can function as antioxidants and lipid protectors in tobacco leaves. Plant Physiol. 147, 764–778 (2008). 18441223 10.1104/pp.108.117614 PMC2409017

[b28] Naqvi S. . Simultaneous expression of Arabidopsis p-hydroxyphenylpyruvate dioxygenase and MPBQ methyltransferase in transgenic corn kernels triples the tocopherol content. Transgenic Res. 20, 177–181 (2011). 20401738 10.1007/s11248-010-9393-6

[b29] Ren W. . Molecular cloning and characterization of 4-hydroxyphenylpyruvate dioxygenase gene from Lactuca sativa. J Plant Physiol. 168, 1076–1083 (2011). 21349599 10.1016/j.jplph.2010.12.017

[b30] Xiao Y. . Characterization and expression profiling of 4-hydroxyphenylpyruvate dioxygenase gene (Smhppd) from Salvia miltiorrhiza hairy root cultures. Mol Biol Rep. 36, 2019–2029 (2009). 19011990 10.1007/s11033-008-9413-2

[b31] Tsegaye Y., Shintani D. & DellaPenna D. Overexpression of the enzyme p-hydroxyphenolpyruvate dioxygenase in arabidopsis and its relation to tocopherol biosynthesis. Plant Physiol. Biochem. 40, 913–920 (2002).

[b32] Chrost B., Falk J., Kernebeck B., Mölleken H. & Krupinska K. In The Chloroplast: From Molecular Biology to Biotechnology (eds Argyroudi-Akoyunoglou Joan H. & Senger Horst) 171–176 (Springer, Netherlands, 1999).

[b33] Valentin H. E. . The Arabidopsis vitamin E pathway gene5-1 mutant reveals a critical role for phytol kinase in seed tocopherol biosynthesis. Plant Cell. 18, 212–224 (2006). 16361393 10.1105/tpc.105.037077 PMC1323494

[b34] Vom Dorp K. . Remobilization of Phytol from Chlorophyll Degradation Is Essential for Tocopherol Synthesis and Growth of Arabidopsis. Plant Cell. 27, 2846–2859 (2015). 26452599 10.1105/tpc.15.00395 PMC4682318

[b35] Collakova E. & DellaPenna D. The role of homogentisate phytyltransferase and other tocopherol pathway enzymes in the regulation of tocopherol synthesis during abiotic stress. Plant Physiol. 133, 930–940 (2003). 14512521 10.1104/pp.103.026138 PMC219066

[b36] Cahoon E. B. . Metabolic redesign of vitamin E biosynthesis in plants for tocotrienol production and increased antioxidant content. Nat Biotechnol. 21, 1082–1087 (2003). 12897790 10.1038/nbt853

[b37] Bassel G. W. . Elucidating the germination transcriptional program using small molecules. Plant Physiol. 147, 143–155 (2008). 18359847 10.1104/pp.107.110841 PMC2330302

[b38] Sen C. K., Khanna S., Roy S. & Packer L. Molecular basis of vitamin E action. Tocotrienol potently inhibits glutamate-induced pp60(c-Src) kinase activation and death of HT4 neuronal cells. J Biol Chem. 275, 13049–13055 (2000). 10777609 10.1074/jbc.275.17.13049

[b39] Nobata Y. . Alpha-Tocopherol Inhibits IL-8 synthesis induced by thrombin and high glucose in endothelial cells. Horm Metab Res. 34, 49–54 (2002). 11972286 10.1055/s-2002-20523

[b40] Yamauchi J. . Tocopherol-associated protein is a ligand-dependent transcriptional activator. Biochem Biophys Res Commun. 285, 295–299 (2001). 11444841 10.1006/bbrc.2001.5162

[b41] Chen P.-Y., Wang C.-K., Soong S.-C. & To K.-Y. Complete sequence of the binary vector pBI121 and its application in cloning T-DNA insertion from transgenic plants. Molecular Breeding. 11, 287–293 (2003).

[b42] Clough S. J. & Bent A. F. Floral dip: a simplified method for Agrobacterium-mediated transformation of Arabidopsis thaliana. Plant J. 16, 735–743 (1998). 10069079 10.1046/j.1365-313x.1998.00343.x

[b43] Chen T. . Expression of an alfalfa (Medicago sativa L.) ethylene response factor gene MsERF8 in tobacco plants enhances resistance to salinity. Mol Biol Rep. 39, 6067–6075 (2012). 22209951 10.1007/s11033-011-1421-y

[b44] Livak K. J. & Schmittgen T. D. Analysis of relative gene expression data using real-time quantitative PCR and the 2(-Delta Delta C(T)) Method. Methods. 25, 402–408 (2001). 11846609 10.1006/meth.2001.1262

[b45] Walley J. W. . The chromatin remodeler SPLAYED regulates specific stress signaling pathways. PLoS Pathog. 4, e1000237 (2008). 19079584 10.1371/journal.ppat.1000237 PMC2588541

[b46] Jefferson R. A., Kavanagh T. A. & Bevan M. W. GUS fusions: beta-glucuronidase as a sensitive and versatile gene fusion marker in higher plants. EMBO J. 6, 3901–3907 (1987). 3327686 10.1002/j.1460-2075.1987.tb02730.x PMC553867

[b47] Fu J., Chu J., Sun X., Wang J. & Yan C. Simple, rapid, and simultaneous assay of multiple carboxyl containing phytohormones in wounded tomatoes by UPLC-MS/MS using single SPE purification and isotope dilution. Anal Sci. 28, 1081–1087 (2012). 23149609 10.2116/analsci.28.1081

