# Supplementary material for: P-HYDROXYPHENYLPYRUVATE DIOXYGENASE from Medicago sativa is involved in vitamin E biosynthesis and abscisic acid-mediated seed germination
Source: Sci Rep. 2017 Jan 13;7:40625. doi: 10.1038/srep40625 (PMC5233959; doi:10.1038/srep40625)

P-HYDROXYPHENYLPYRUVATE DIOXYGENASE from *Medicago sativa* is involved in vitamin E biosynthesis and abscisic acid-mediated seed germination

Jishan Jiang[1,4], Zhihong Chen[2], Liping Ban[3], Yudi Wu[1], Jianping Huang[1,3], Jinfang Chu[5], Shuang Fang[5], Zan Wang[1], Hongwen Gao[1*], Xuemin Wang[1*]

1. Institute of Animal Science, Chinese Academy of Agricultural Sciences, Beijing 100193, China.
2. National Animal Husbandry Service, Ministry of Agriculture, Beijing 100125, China.
3. College of Animal Science and Technology, China Agricultural University, Beijing 100193, China.
4. Present address: Department of Botany and Plant Sciences, University of California, Riverside, CA, 92521.
5. National Centre for Plant Gene Research, Institute of Genetics and Developmental Biology, Chinese Academy of Sciences, Beijing 100101, China.

* Corresponding author: E-mail: wangxuemin@caas.cn; gaohongwen@263.net

Supplemental Figure legend

Supplemental Fig. 1 *cis*-elements presented in the promoter region of *MsHPPD*.

Supplemental Fig. 2 Vitamin E profiles in MsHPPD-overexpressing lines and wild type Arabidopsis seeds and leaves. A: seeds; B: leaves
Seeds of transgenic and control Arabidopsis were harvested at the same time, dried at room temperature for two weeks, and used for measurement in (A). Two-week-old Arabidopsis leaves were used for measurement in (B). Data are presented as mean ±SD using three biological replicates.

Supplemental Fig. 3 Potential model for *MsHPPD* to regulate seed germination.

Supplemental figure 1

Cis-elements	Sequences	Function	No.
ACE	ACGTGGA	Involved in light responsiveness	1
ARE	TGGTTT	Essential for the anaerobic induction	2
AT1-motif	AATTATTTTTTATT	Part of a light responsive module	1
ATC-motif	AGTAATCT	Involved in light responsiveness	1
Box 4	ATTAAT	Involved in light responsiveness	2
G-Box	CACGTT	Involved in light responsiveness	2
G-box	CACGAC/CACGTT	Involved in light responsiveness	4
GA-motif	AAGGAAGA	Part of a light responsive element	1
GARE-motif	AAACAGA	Gibberellin-responsive element	1
GATA-motif	AAGGATAAGG	Part of a light responsive element	1
LAMP-element	CCTTATCCA	Part of a light responsive element	1
MRE	AACCTAA	MYB binding site involved in light responsiveness	1
Skn-1_motif	GTCAT	Required for endosperm expression	3
TC-rich repeats	ATTCTCTAAC	Involved in defense and stress responsiveness	1
TCA-element	GAGAAGAATA	Involved in salicylic acid responsiveness	1
TCT-motif	TCTTAC	Part of a light responsive element	3
TGA-element	AACGAC	Auxin-responsive element	1
Circadian	CAANNNNATC	Involved in circadian control	1

Supplemental figure 2

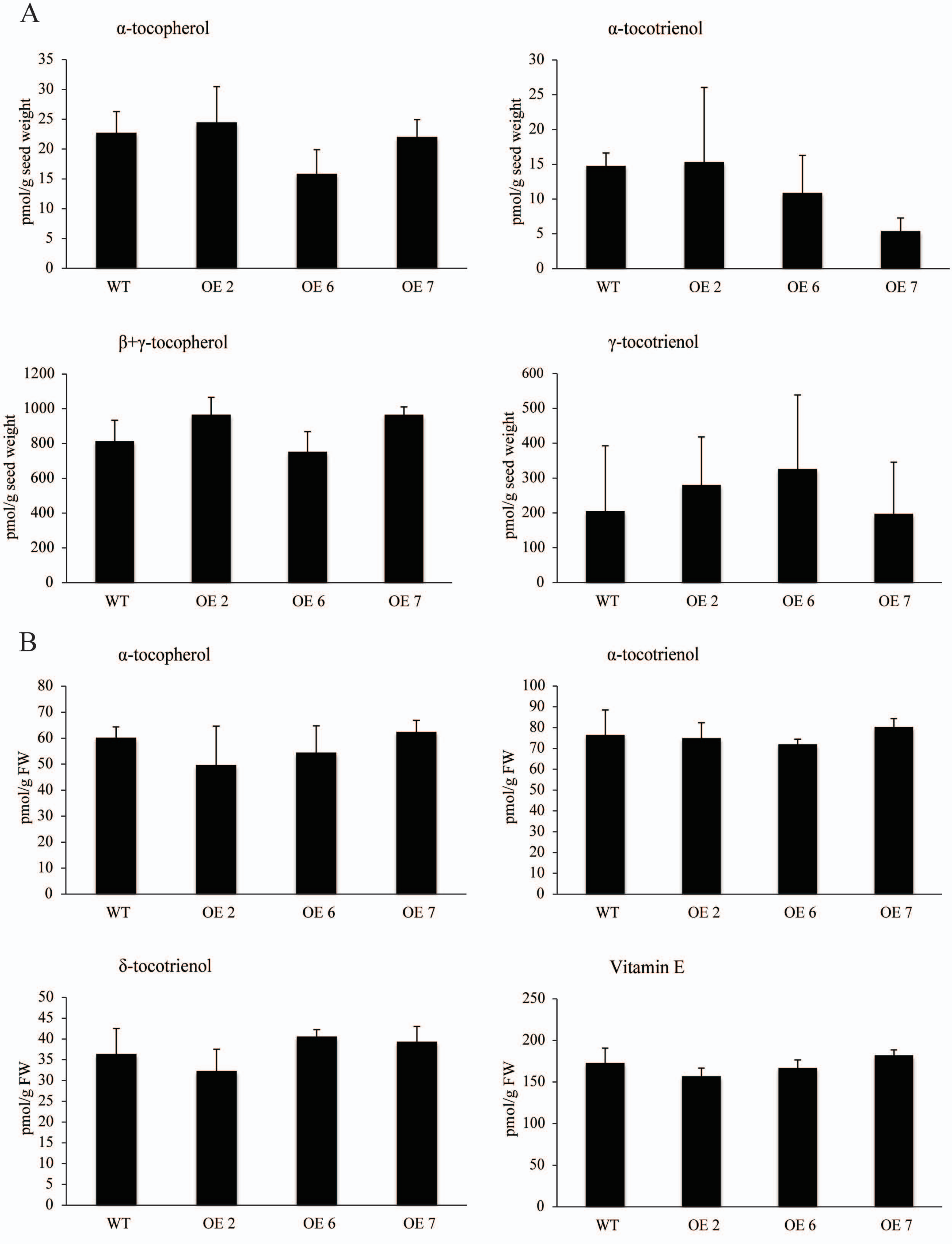

Supplemental figure 3

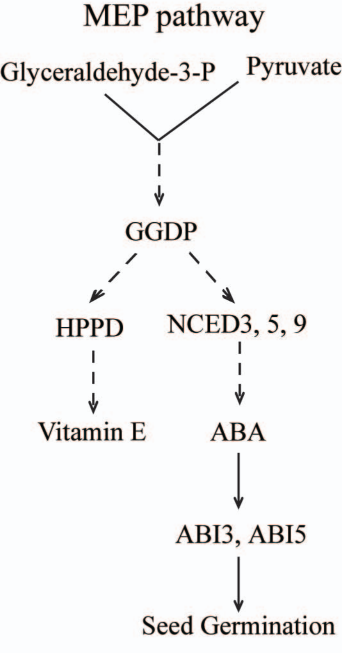

Supplement: Supplementary Information [file srep40625-s1.pdf]
